# The importance of muscle strength and physical performance as part of the diagnosis and management of sarcopenia in young adults living with human immunodeficiency virus

**DOI:** 10.20945/2359-4292-2025-0018

**Published:** 2025-10-08

**Authors:** Bárbara Gehrke, Maria Lucia Fleiuss Farias, Luiz Eduardo Wildemberg, Giovanna Ianini Ferraiuoli, Valéria Ribeiro, Rogério Bosgnoli, Francisco de Paula Paranhos Neto, Laura Maria Carvalho de Mendonça, Miguel Madeira, Maria Caroline Alves Coelho

**Affiliations:** 1 Programa de Pós-graduação em Fisiopatologia Clínica e Experimental, Universidade Estadual do Rio de Janeiro, Rio de Janeiro, RJ, Brasil; 2 Centro de Pesquisa Clínica Multiusuário, Universidade Estadual do Rio de Janeiro, Rio de Janeiro, RJ, Brasil; 3 Divisão de Endocrinologia, Departamento de Medicina Interna, Faculdade de Ciências Médicas, Universidade Estadual do Rio de Janeiro, Rio de Janeiro, RJ, Brasil; 4 Divisão de Endocrinologia, Universidade Federal do Rio de Janeiro, Rio de Janeiro, RJ, Brasil; 5 Divisão de Neuroendocrinologia, Instituto Estadual do Cérebro Paulo Niemeyer, Secretaria Estadual de Saúde, Rio de Janeiro, RJ, Brasil; 6 Divisão de Infectologia, Hospital Universitário Pedro Ernesto, Universidade do Estado do Rio de Janeiro, Rio de Janeiro, RJ, Brasil; 7 Labhor - Laboratório de Hormônios da Endocrinologia, Hospital Universitário Pedro Ernesto, Universidade do Estado do Rio de Janeiro, Rio de Janeiro, RJ, Brasil; 8 Divisão de Reumatologia, Departamento de Medicina Interna, Universidade Federal do Rio de Janeiro, Rio de Janeiro, RJ, Brasil

**Keywords:** Body composition, sarcopenia, muscle strength, appendicular lean mass, HIV

## Abstract

**Objective:**

To evaluate muscle functionality, physical performance and body composition in young people living with human immunodeficiency virus (PLWH).

**Subjects and methods:**

Eighty-one HIV-infected and 54 uninfected (20 to 50 years) male and female subjects were enrolled to participate. Patient evaluation included body composition by DXA (dual energy X-rays), SARC-F questionnaire, hand grip and timed up & go (TUG) tests.

**Results:**

Fifty PLWH and 50 age-gender matched controls completed the study. The median age was 40 (25-49) vs. 36.5 (22-50) for the HIV and control groups, respectively (p 0.120). Race, gender, body mass index, phosphorus and 25-hydroxyvitamin D were similar between groups. HDL-c was significantly lower in HIV-infected (p 0.006). Groups had similar body composition parameters, although more PLWH presented appendicular lean mass (ALM) and ALM adjusted to height (ALM/h^2)^ below reference values (18% vs 4%). SARC-F questionnaire and TUG were significantly compromised in HIV-infected when compared to controls (p 0.001 and 0.005, respectively). Hand grip test was slightly lower in PLWH than in control group (29.0 kg (9.3-56.0) vs. 32.8 kg (13.3-57.3); p 0.052).

**Conclusion:**

Our results suggest that there is loss of functionality, physical performance and muscle strength in young PLWH. Therefore, screening using SARC-F, hand grip and TUG test might be interesting in HIV-infected which are considered at high-risk for sarcopenia. With early diagnosis there is the possibility of decreasing muscle dysfunction, morbimortality, providing an increase in quality of life and working hours.

## INTRODUCTION

People living with human immunodeficiency virus (PLHIV), nowadays, present higher life expectancy thanks to the development of antiretroviral therapies (ART). This population have almost the same life expectancy as healthy individuals and they may develop chronic diseases, such as osteoporosis, cardiovascular disease, diabetes, and sarcopenia more frequently ^(1)^. The prevalence of sarcopenia in PLHIV is around 30% and this number is similar to the prevalence in individuals with cardiovascular disease, dementia, diabetes mellitus and respiratory disease in the general population ^(1,2)^.

Sarcopenia consists of a generalized and progressive skeletal muscle disease that may culminate in adverse outcomes such as: reduced functionality and physical performance and higher risk of falls and fractures, therefore, increased morbimortality ^(3)^. Sarcopenia is frequent in elderly individuals, but it may be recognized in earlier stages of life, mainly in people with risk factors and comorbidities. There is evidence in literature that young PLHIV present higher prevalence of sarcopenia when compared to healthy controls, which may vary between 17.5 and 21.9% in women and men, respectively ^(4,5)^.

The period of highest muscle strength is observed in healthy individuals during young adulthood (up to 40 years), naturally lower in women when compared to men ^(6)^. Muscle strength decline occurs around 16.6 to 40.9% in > 40 years when compared to younger population ^(6)^. The diagnosis of sarcopenia is identified by low muscle strength and confirmed by low muscle quantity or quality. These two associated with low physical performance classify sarcopenia as severe ^(3)^.

There are several risk factors that contribute for the development of sarcopenia in PLHIV such as use of ART and chronic immune activation caused by virus leads to a low-grade systemic inflammation (LGSI - low grade systemic inflammation), which can reduce the activity of substances responsible for the synthesis of muscle proteins and lead to a dysfunction of the adipose tissue, with ectopic fat accumulation. Antiretrovirals promote mitochondrial damage through increased muscle inflammation and, by direct action, can reduce gene expression of proteins involved in protein synthesis ^(7)^. In addition, they can lead to changes in body composition.

There is also evidence that Receptor Activator of Nuclear Factor kappa-B Ligand (RANKL) plays an important role in the inhibition of myogenic differentiation. PLHIV present increased expression of RANKL, favoring bone and muscle loss and dysfunction ^(8)^.

The objective of our study is to comprehend the different tools that may be used to diagnose sarcopenia in young PLHIV.

## SUBJECTS AND METHODS

### Study design and population

A cross-sectional study was performed, and 135 participants (81 PLHIV and 54 uninfected) were recruited to participate. The study was carried out during the period of March 2020 and April 2023. Inclusion criteria were men and women with age between 20 and 50 years. All participants signed written informed consent and the study’s ethical standards were in accordance with the Helsinki Declaration. Study protocol was approved by the Research Ethics Committee of Hospital Universitário Pedro Ernesto (HUPE/State University of Rio de Janeiro, Brazil) - number 29162020.1.0000.5259.

PLHIV were enrolled from an outpatient clinic for infectious diseases at HUPE. These participants needed to have the diagnosis of HIV infection for at least one year, could be treating with ART or not, and needed to attend medical appointments at least every six months. Data concerning infection and treatment were gathered in HIV-infected subjects. Healthy control participants were recruited from the internal medicine outpatient clinic at HUPE.

Exclusion criteria were menopause or premature ovarian failure (women with irregular menstrual cycles), low testosterone levels in males (total testosterone below 8 nmol/L or free testosterone levels below 0.225 nmol/L) ^(9)^, glomerular filtration rate below 60 mL/min/1.73 m^2^, weight superior to 120 kilograms (kg) (due to equipment maximum capacity), pregnancy, use of medications that may interfere with muscle and bone health (chronic use of glucocorticoids, anticonvulsants, proton pump inhibitors, loop diuretics) and preexisting conditions that may affect muscle and bone metabolism (endocrine, liver, kidney, rheumatologic and hematologic diseases, tubular renal acidosis, malabsorptive intestinal syndromes or bariatric surgery, and neoplasms).

### Biochemistry

Blood was collected during fast for routine tests (hemogram, glucose, glycated hemoglobin, lipid profile, urea, creatinine, alkaline reserve, hepatic function), free T4, TSH, 25 hydroxyvitamin D ^(10)^ and sexual hormones. Morning total testosterone, serum albumin and sex-hormone-binding globulin (SHBG) levels were measured in all male subjects. Free testosterone was calculated according to the Vermeulen formula ^(11,12)^. 25(OH)D was measured by competitive chemiluminescence (Maglumi X8 from Snibe, China). Recommended levels were ≥ 20 ng/mL for healthy controls and ≥ 30 ng/mL for those at risk of fractures, which include PLHIV ^(10)^.

### Physical exam

Anthropometric data was collected, such as: weight (kg), height (m) and body mass index (BMI - calculated by the reason of weight over squared height in kg/m^2)^.

### Evaluation of muscle parameters and performance

As detailed below, lean mass was evaluated by dual energy X-ray absorptiometry (DXA) whereas muscle function and performance were evaluated by the SARC-F questionnaire, hand grip test and timed up & go (TUG), as recommended by the European Working Group on Sarcopenia in Older People (EWGSOP2) ^(3)^.

#### SARC-F questionnaire

SARC-F consists of a self-reported questionnaire used for screening of sarcopenia risk. The Portuguese language version of the questionnaire was used ^(13)^. Patients that score ≥ 4 are likely to have sarcopenia, according to literature, in elder individuals ^(3,14)^.

#### Timed Up & Go (TUG)

This test evaluates the participant’s physical performance. The time taken to complete the movement is considered altered when > 20 seconds for elder individuals ^(15)^.

#### Hand Grip Test

The hand grip test evaluates muscle strength. Each participant exerted their highest muscular strength capacity with dominant arm using a handheld calibrated manual dynamometer (Jamar Model 1 - 70729, Asimow Engineering Company, Santa Monica, CA, USA), according to the recommendations of the American Association of Hand Therapists ^(16)^. Grip strength is considered weak when < 26 kg for men and < 16 kg for women, determined by the Foundation for the National Institutes of Health (FNIH) Sarcopenia Project ^(17)^. Intermediate grip strength is classified between 26-31.9 kg in men and 16-19.9 kg in women ^(17)^.

### Body composition by DXA

The evaluation of lean mass was performed by DXA with the objective of determining muscle quantity. Lean mass measured at upper and lower limb was used to calculate appendicular lean mass (ALM) in kg. ALM was then adjusted to height by calculating the reason of ALM and squared height (kg/m^2)^ ^(18)^. Cut-offs suggested by the EWGSOP2 and based on Baumgartner’s operational definition were used, which correspond to > 2 standard deviations (SD) below the mean reference values for young controls with ages between 18 and 40 years (Z score), in which abnormal results for appendicular lean mass index (ALMI = ALM/height^2)^ are <5.5 kg/m^2^ and < 7.00 kg/m^2^ and ALM are < 15 kg and < 20 kg, for women and men, respectively ^(3,19,20)^. Fat, lean and tissue mass in grams (g); fat region and fat tissue (%); visceral adipose tissue and subcutaneous adipose tissue area, volume, and mass (cm^2^, cm^3^ and g, respectively) were also analyzed ^(21)^.

### Diagnosis of sarcopenia

According to the recommendations of EWGSOP2, the criterion used to classify as probable sarcopenia was low muscle strength (altered hand grip test) and diagnosis of sarcopenia is confirmed by low muscle quantity (low appendicular lean mass by DXA). The condition is considered severe when both criteria are achieved in association with low physical performance (altered TUG) ^(3)^.

### Statistical analysis

SPSS version 29.0.0.0 (241) for Windows (SPSS Inc., Chicago, IL, USA) was used for statistical analysis. Non-parametric tests were used as the Kolmogorov-Smirnov test revealed that most numerical variables didn’t respect normality curve. The study compared results between healthy subjects and PLHIV. Chi-squared test or Fisher’s test were used to compare categoric variables. Numerical variables used Mann-Whitney or Student t tests. Correlations between numeric variables were evaluated by Pearson or Spearman tests. Strength degrees of correlation were based on correlation coefficient (*r*): very weak (0.00-0.19), weak (0.20-0.39), moderate (0.40-0.69), strong (0.70-0.89) and very strong (0.90-1.00). Differences were considered statistically significant when α = 5% (p <0.05).

## RESULTS

Medical records of 135 young PLHIV on current treatment at our institution were evaluated. After signed consent, they were submitted to laboratory tests, in which abnormalities leaded to exclusion (four controls were excluded due to hypogonadism or diabetes, 6 PLHIV were excluded due to chronic glucocorticoid use/hospitalization, hypogonadism or diabetes, while 25 PLHIV lost follow-up) and scheduled for physical function tests and DXA. Fifty PLWH and 50 age-gender matched controls completed the study with SARC-F questionnaire, TUG, hand grip test and body composition.

PLHIV consisted of 24 women and 26 men, median age 40 years (25-49). Duration of HIV infection was estimated as 156 (36-480) months, exposure to tenofovir disoproxil fumarate (TDF) was 96 (0-252) months, and serum CD4+ was 659.5 (65-1,279) cell/mm^3^.

Groups were similar in respect to age, race, gender, BMI, serum phosphorus and vitamin D levels. The only metabolic difference was a lower HDL-cholesterol (HDL-c) in HIV-patients. Median BMI was compatible with overweight in both groups. However, PLHIV performed worse on physical tests, as shown in **Table 1**.

**Table 1:** Participants demographic and anthropometric characteristics, as well as biochemistry results and muscle function tests, in human immunodeficiency virus group and matched controls

	HIV group n = 50	Control group n = 50	p
Gender	24 F/26 M	25 F/25 M	1.00
Ethnicity (% Caucasian)	56	58	1.00
Age (years)	40 (25-49)	36.5 (22-50)	0.120
Weight (kg)	76.7 (50.1-102.6)	76.7 (54.4-109.8)	0.448
Height (cm)	166 (150-187)	169.5 (153-186)	0.473
BMI (kg/cm^2)^	26.9 (17.44-37.5)	27.7 (19.34-39.4)	0.459
Biochemistry			
Serum phosphorus (mg/dL)	3.8 (2.7-5.8)	3.9 (2.9-5.4)	0.568
25(OH) vitamin D (ng/mL)	27.7 (12.9-42.4)	27.9 (13.5-57.8)	0.936
25(OH)D below 30 ng/mL (%)	58	56	1..000
HDLc (mg/dL)	44.5 (23-124)	53 (19-99)	0.006
LDLc (mg/dL)	110.8 (56-177.2)	113 (76.6-202)	0.146
Triglycerides (mg/dL)	103 (37-405)	81 (21-276)	0.189
Muscle Function Tests			
Test Get Up & Go (seconds)	8.91 (5.70-16.26)	8.23 (5.57-10.95)	0.005
Grip test (kg)	29.0 (9.3-56.0)	32.8 (13.3-57.3)	0.052
SARC-F (score)	0 (0-4)	0 (0-2)	0.001

Variables are expressed in median values (minimum and maximum).

HIV: Human immunodeficiency virus; F: female; M: male; 25 (OH) D: 25-hydroxi-vitamin D; HDLc: high density lipoprotein; LDLc: low density lipoprotein.

In PLHIV group 16 participants (32%) took more than 10 seconds to complete TUG test, whilst in control group there were only 3 (6%). None of the participants of the study (in both groups) took more than 20 seconds to complete the test.

**Table 2** presents body composition results obtained with DXA. All analyzed parameters were similar between groups either those related to fat mass distribution and visceral fat, or those related to muscle mass.

**Table 2:** Body composition results by dual energy X-ray absorptiometry (DXA) in human immunodeficiency virus-infected and matched control participants

	HIV group n = 50	Control group n = 50	p
Total mass (kg)	76.7 (50.1-102.6)	76.7 (54.4-109.8)	0.448
Total tissue mass (g)	74252.5 (47901-100127)	74321 (52588-107069)	0.448
Total lean mass (g)	47569 (33215-69460)	50085 (33777-71506)	0.234
Total fat free mass (g)	50233.5 (35302-72931)	52580 (35588-75869)	0.236
ALM (kg)	22.55 (13.8-32.8)	24.3 (13.5-35.4)	0.198
ALM/h^2^ (kg/m^2)^	7.95 (5.51-10.63)	8.42 (5.36-10.82)	0.137
ALM/BMI (kg/kg/m^2)^	0.79 (0.494-1.217)	0.83 (0.531-1.238)	0.477
Total fat mass (g)	24140.5 (11923-53610)	26030 (9362-54687)	0.845
Total fat region (%)	32.75 (18.5-52.3)	33.5 (14.6-49.8)	0.767
Total fat tissue (%)	34 (19.3-53.5)	34.8 (15.2-51.1)	0.751
VAT volume (cm^3)^	600 (103-2368)	622 (2-1995)	0.374
VAT mass (g)	566 (97-2234)	586.5 (2-1882)	0.374
VAT area (cm^2)^	71 (14-257)	69.5 (0-235)	0.372
SAT volume (cm^3)^	1401 (51-3579)	1492 (152-3737)	0.836
SAT mass (g)	1322 (48-3376)	1408 (144-3525)	0.836
SAT area (cm^2)^	168 (6-430)	165 (16-401)	0.803

kg: kilograms; G: grams; ALM: Appendicular lean mass; ALM/h^2^: Appendicular lean mass adjusted for height squared; ALM/BMI: Appendicular lean mass adjusted for body mass index; VAT: Visceral adipose tissue (intra-abdominal fat); SAT: Subcutaneous adipose tissue; cm^2^: squared centimeters; cm^3^: cubic centimeters.

Five (10%) of PLWH presented intermediate grip strength while seven (14%) evidenced weak grip strength. Therefore, seven (14%) of our HIV-infected sample presented probable sarcopenia. Two percent of PLHIV had sarcopenia (weak hand grip plus low muscle mass). In control group one participant (2%) presented weak grip strength classifying as probable sarcopenia. None of the control subjects confirmed diagnosis of sarcopenia with concomitant low muscle quantity. The lowest value of muscle strength for women in HIV group was 9.3 kg versus 13.3 kg for a woman in control group.

The population was studied for statistical correlations for all variables analyzed in the study. The parameters that showed significant correlations are exposed in **Table 3**.

**Table 3:** Correlations between time of infection, CD4+ cell count and exposure to tenofovir disoproxil fumarate with muscle function tests and body composition results in human immunodeficiency virus-infected group

	Time of infection	CD4+ cell count	Exposure to TDF
TUG (seconds)	p 0.029; r 0.309	---	---
Total fat tissue (%)	p 0.005; r 0.393	---	p 0.015; r 0.343
Total fat region (%)	p 0.006; r 0.386	---	p 0.018; r 0.334
VAT area (cm^2)^	p 0.034; r 0.309	---	---
Hand grip test (kg)	p 0.037; r -0.276	p 0.030; r 0.307	---
Total lean mass (g)	p 0.002; r -0.420	---	p 0.026; r -0.315
ALM (kg)	p <0.001; r -0.452	---	p 0.028; r -0.310
ALM/h^2^ (kg/m^2)^	p 0.045; r -0.285	---	---
ALM/BMI (kg/kg/m^2)^	---	---	p 0.014; r -0.346

TDF: tenofovir disoproxil fumarate; TUG: Test Up & Go; VAT: Visceral adipose tissue (intra-abdominal fat); cm^2^: squared centimeters; kg: kilograms; G: grams; ALM: Appendicular lean mass; ALM/h^2^: Appendicular lean mass adjusted for squared height.

## DISCUSSION

Considering that frequently used definitions for diagnosis of sarcopenia are mainly for elderly populations, the results found are substantial for a younger group. The use of functional muscle tests to screen for muscle disorders and progressive loss of functionality in young PLHIV are of extreme importance. The fact that in literature there are only cut-off values that contemplate older populations, highlight the need of new protocols focused on younger individuals. Also, more studies are needed to understand the role of ALM and ALM/h^2^ in body composition assessment of young HIV-infected. The current study is the first to evaluate muscle strength, functionality and physical performance as part of sarcopenia’s diagnosis in young PLWH.

SARC-F questionnaire was applied to screen sarcopenia and data evidenced a statistically significant difference between groups, considering altered results whenever subjects scored ≥ 1, with higher values for PLHIV. This underscores that PLWH may develop physical limitations and higher morbidity when compared to healthy individuals with similar age. However, of the 24% from HIV-infected group that presented altered results for hand grip strength, only 10% had at least one concomitant mobility or physical functionality complaint while answering the questionnaire. For this reason, SARC-F questionnaire might not be the best tool for initial screening of sarcopenia in young PLHIV, as it may underestimate diagnosis considering its focus on elderly individuals ^(22)^.

Also, higher TUG results in PLWH, may indicate a compromise of mobility and physical performance in young PLHIV.

In reference to muscle strength evaluation, 14% of PLHIV had probable sarcopenia while only 2% in control group. EWGSOP2 highlight that low muscle strength as a determinant of sarcopenia diagnosis has gained force recently, outweighing the importance of muscle mass at diagnosis ^(3)^. Also, they concluded that although sarcopenia is associated with decreased muscle quantity and quality, muscle strength is better than the quantification of muscle mass to predict undesirable outcomes ^(3)^.

Results from this study suggest decreased functionality and mobility in young PLHIV, which may be related to lower appendicular lean mass. Although median values of body composition parameters did not differ between PLHIV and controls, the proportion of PLWH with ALM and ALM/h^2^ below reference values was higher than that found in controls, although not statistically significant. Higher BMI, serum CD4 values and proportion of individuals in ART are factors that may attenuate the difference of muscle mass in PLHIV when compared to controls ^(2)^.

Several studies have shown the association of PLHIV and ART with low muscle mass, sarcopenia and fat redistribution ^(5,7,23-25)^. A systematic review and metanalysis of 2022 concluded that the prevalence of sarcopenia in 18 years or older (mean ages from 33.0 to 62.1 and from 24.2 to 60 in PLWH and healthy controls, respectively) is 30.3% for low muscle mass only and 4.5% for low muscle mass and strength in PLHIV ^(2)^. The age ranges from the latter study also considered individuals over 50 years, therefore, a larger number of individuals with low muscle mass was found when compared to our study (30.3% vs. 18%). Also, using the same criteria, low muscle mass and strength were identified in 2% of our HIV sample against 4.5% in this metanalysis that considers a higher age range.

Konishi and cols. investigated sarcopenia in Japanese PLHIV (male subjects > 60 years) and found that 10.3% had pre sarcopenia (low muscle mass) and 16.1% had sarcopenia (low muscle mass and low muscle strength or decreased physical function) ^(26)^. In our study, 18% evidenced pre sarcopenia and 2% had sarcopenia. These differences might be explained by the differing age ranges and race of each study.

Regarding the use of TDF, cases of renal tubulopathy with hypophosphatemia have been described in PLHIV ^(27-29)^. Nevertheless, in our sample none of the HIV-infected presented hypophosphatemia.

The longer duration of HIV-infection is related to fat accumulation and muscle loss, as well as increase in time at TUG (reduced physical performance) and decrease in muscle strength. Higher CD4^+^ values were associated to higher grip strength, which means that immunocompetent PLHIV were able to maintain muscle strength. Also, longer exposure to TDF was correlated to muscle loss and fat accumulation. Although correlations were statistically significant, most coefficients were weak. Nevertheless, longer periods of HIV-infection, ART exposure and immunodeficiency must draw attention to unfavorable outcomes related to sarcopenia and these findings are compatible with literature ^(22)^. No significant correlations were found regarding other ART regimens.

Nowadays the available options to treat sarcopenia are mainly non-pharmacological ^(30)^. Literature suggests that vitamin D status must always be assessed [recommended levels ≥ 30 ng/dL for HIV-infected ^(10)^], as well as and protein intake [1.0-1.2 g/kg body weight per day divided in several meals ^(31)^] to provide adequate recommendations as preventive measures. Also, healthcare professionals must incentive the practice of ≥ 150 minutes per week of physical activity divided in aerobic and resistance exercises ^(32)^. These measures may prevent not only sarcopenia and osteoporosis, but also weight gain and metabolic disorders in PLWH. Physical activity plays an important role in the preservation of muscle strength and physical functionality according to literature ^(22,30)^.

A limitation of our study was the impossibility of measuring muscle quality. Magnetic resonance imaging and computed tomography have been used to determine muscle attenuation and fat infiltration, but their use has been limited to research purposes as there is no cut-off values defined for the diagnosis of sarcopenia ^(21,33)^. Also, there was no evaluation of protein intake, which is an important determinant of muscle health.

Sarcopenia is still underdiagnosed in young PLHIV, which are considered at high-risk. Muscle function tests should be considered in this group of individuals to reach a higher number of patients with functionality and mobility commitment that might benefit from sarcopenia treatment. Factors such as: duration of disease, exposure to ART and immunodeficiency seem to be important determinants of muscle dysfunction and require attention. If not treated, sarcopenia may culminate in dependence, reduced physical performance, higher morbimortality, reduction of working capacity and of quality of life. Thus, this study draws attention to the importance of investigating sarcopenia since disease onset, covering every aspect of this individual’s treatment to provide long-lasting health.

## Data Availability

datasets related to this article will be available upon request to the corresponding author.
